# Slack

**DOI:** 10.5195/jmla.2018.315

**Published:** 2018-01-02

**Authors:** Heather A. Johnson

## INTRODUCTION

It is not uncommon for librarians to collaborate across institutions, especially when organizing events such as annual meetings. In these cases, organizing committees may have representation from across multiple states and organizations, and therefore, in-person communication may not always be possible. Therefore, it is useful for teams to use collaborative tools that facilitate easy, streamlined communication. In some cases, email may be sufficient for brief, infrequent communications, but when a team comprises more than a few people discussing a number of topics, email may impede productivity.

Email may become burdensome when the team creates a high volume of messages that must be opened and read in the context of either the original reply or someone else’s reply, and the reader must distinguish which reply is in response to which email. Email may also pose a problem when there are multiple synchronous discussions occurring. In this case, it may be difficult to immediately determine which topic is being discussed. Lastly, email may be unreliable when searching for specific conversations. Emails may be subject to spontaneous deletion or archiving, or content may be too broad to search effectively. To combat these three problems, team leaders should identify a solution that confines communications to one place, separates topical conversations, and archives communications, making conversations searchable and teams more productive and efficient.

Slack is a cloud-based digital workspace and information management system used to manage productivity and improve team efficiency. In a recent survey of Slack administrators, 32.0% of respondents noted that Slack increased their productivity, reducing internal email by 48.6% and meetings by 25.1% [1]. Further, 79% of respondents felt that Slack helped to improve the culture of their teams, with 88.6% of administrators indicating they felt more connected to their teams. In terms of information management, 80.4% of respondents cited improved transparency within the team environment, while 62.4% of survey respondents believed that Slack made it easier for them to locate information.

Slack has several pricing tiers, but this review will focus on the free version of the product.

## AUDIENCE

This product (free or otherwise) would be most useful for individuals working with a team to complete a project with several components. I tested this product while planning the 2018 North Atlantic Health Sciences Libraries (NAHSL) annual meeting. The committee comprised seventeen members from thirteen locations across four states. Committee members belonged to one of twelve subcommittees. With several subcommittees, each with several committee members, there were naturally several conversations at the same time. With Slack, we created a number of channels (described below in detail) specific to each major conversational theme, allowing us to have a number of concurrent conversations while keeping each conversation separate.

## MAJOR FEATURES

Among the many useful features of Slack are those related to accessibility, streamlined communication, content organization, and platform integration. Slack is broadly accessible, in that members can access their workspaces via the web, a desktop plugin, or a mobile application, and compared to other products, it was the least likely to be blocked by an institutional firewall.

Slack also provides a number of options for members to organize content, first by channel, and more granularly by way of posts and cloud-based document storage. The platform also allows streamlined communication that transcends email reduction. It allows broad communication via real-time chat, as well as direct, private messaging. Slack also archives all content posted to public domains (i.e., not direct message), making it easily searchable, barring limitations described below. Lastly, Slack integrates with hundreds of native and external applications, serving as a hub for content in project-related platforms such as Google Drive, Trello, and SurveyMonkey.

### Accessibility

For any type of team-based solution to be useful, it must be accessible by all team members, including those from organizations with various levels of security. Our team had varied access with HipChat and Glip, two tools that offered features similar to Slack’s. While team members at academic institutions were able to seamlessly create accounts with HipChat and Glip, colleagues at hospital libraries were not always successful due to firewalls. Of HipChat, Glip, and Slack, Slack was the only tool that was accessible to all team members.

### Workspaces and teams

**Figure 1:**
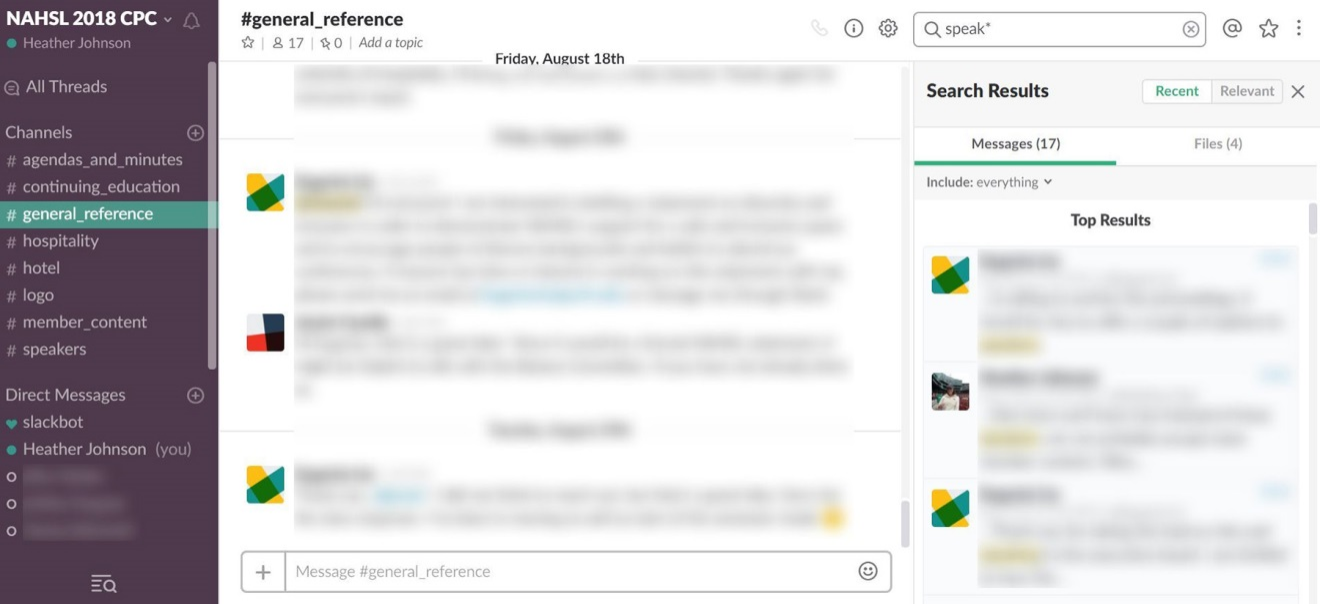
Slack workspace

A Slack workspace (Figure 1) is intended to streamline communications, keeping them in one central location that can be searched and integrated with external applications. All communications—including messages and attachments such as portable document format files (PDFs), Word documents, and Google Docs—are indexed and easily searchable using simple or advanced terms (e.g., contract in:library_remodel; contract).

A workspace comprises channels, which are essentially chat rooms dedicated to a specific group, topic, or project. Team members can use these channels to participate in ongoing conversations. A workspace can take on several forms, depending on the needs of the team owners. A team owner who is a library director may wish to create one workspace to house a number of projects, designating one channel per project (e.g., Workspace: Medical Library Projects; Channels: Collection Development, Systematic Reviews, Remodel).

A team owner can also be a project manager dedicating an entire workspace to a single project, assigning channels specific to different subprojects (e.g., Workspace: Annual Meeting; Channels: Speakers, Hospitality, Branding). Team owners can limit access to certain channels to specific members, or they may grant permission for all team members to access all channels. Uses may leave, mute, and archive channels as needed.

### Organization, archiving, and indexing

Slack keeps communications in one central location, thus reducing email, creating a searchable archive, and organizing messages by topic. Slack helps to reduce email with real-time chat, with which users can contribute to a group conversation, viewable by all team members, without replying to all on an email. Team members can check in on conversations when convenient and avoid being bombarded by email. This may be especially useful for team members who belong to a project team but are not interested in a particular subtopic (e.g., the hotel subcommittee chair may not be interested in branding).

The free version of Slack allows users to search within the last 10,000 messages sent by all members of the team. Once a team reaches the 10,000-message limit, they can continue to send messages and search within the most recent 10,000 messages. While channel-wide and direct message count toward the 10,000 message limit, posts do not. Posts can take 2 forms: to-do lists and long-form collaborative messages that may be shared with a single person or with multiple team members. A post can be useful when 2 people wish to work together to draft an email or edit a contract.

### Integration

Slack integrates with more than 600 native and external applications for file management, analytics, project management, social tools, and more. For file management, such apps include Box, Dropbox, and Google Drive. Slack allows users to import files, making them searchable from within the Slack platform. Team members can also create internal team surveys using Simple Poll, a native application used to create team polls in Slack. Such polls may ask questions such as, “Which logo do you like best? A or B?”

Team owners can also integrate Slack with project management and productivity tools such as Trello to either connect a team or a specific board and send notifications about board updates to a channel or to the team owner. Other useful integrations include GitHub, PayPal, Twitter, and Google Calendar.

### Mobile and desktop accessibility

In addition to its web-based workspace, Slack also has native applications for Mac and Windows desktop, Android, and iOS. The Slack desktop application ensures that Slack is always running on a user’s computer, eliminating the need to remember to sign in to the web-based interface. The web-based interface and the native applications allow users to customize notification settings, which may be specific to channels.

## PRODUCTS AND PRICING

Slack offers two tiers of service: Slack for Teams and Slack Enterprise Grid. Slack for Teams includes 3 levels with different pricing structures: Free, Standard, and Plus. Slack offers an 85% discount on Standard and Plus plans for nonprofits and educational institutions that meet specific criteria.

- Slack for Teams: Slack for teams is intended for teams comprising 3 to 1,000 people working in a single workspace. Slack for Teams is available for free with a basic account and for a fee with Standard and Plus accounts.
  – Free: With the free version, owners of workspaces may invite an unlimited number of team members to join a workspace. Team members can enjoy up to 10 third-party or custom integrations, share 5 GB of storage, and participate in one-on-one voice and video calls. They may also search within the last 10,000 direct or channel-wide messages in a workspace.
  – Standard: Upgrading to a Standard account provides access to 14 upgraded features. Those features include the ability to search for people and channels and an unlimited number of messages. Team members also have access to 10 GB of storage per user, as well as unlimited external application integrations. Furthermore, the Standard option provides a more robust conference space for users through its screen-sharing capabilities and voice and video calling with up to 15 people. Users also have access to priority customer support.
  – Plus: Users purchasing the Plus option have access to all the features of a Standard account, plus single sign-on; 99.99% guaranteed uptime; and the ability for team owners to track and export all communications, regardless of deletion or private message status. Users also have access to 24/7 customer support with a 4-hour response time.
- Slack Enterprise Grid: For organizations needing robust sharing capabilities, Slack offers an Enterprise option. This option gives institutions the ability to create unlimited workspaces, share teams and channels, search across the organization, and store 1 TB of information. Furthermore, Slack Enterprise Grid provides 24/7 customer support with a 2-hour response time.

## OVERALL VALUE

For the purposes of organizing a conference with a committee size of seventeen, the free version of Slack proved sufficient. Although we were only able to integrate up to ten Slack-approved applications, we did not feel constrained by this limit (we integrated Trello, Google Drive, Trello Notifications, and Simple Poll) and were able to bypass the inability to integrate non-approved websites, such as Canva, by simply using the chat feature to send links to external content. Although the free version makes no mention of customer service response time, my personal experience has been positive, with responses within one hour.

Teams can integrate services like Google+ Hangouts and Skype to convene group voice and video calls, but cobbling together too many services may create confusion and negate the utility of a tool that is intended to improve productivity, streamline communication, and store all information in a single location. In this case, teams may benefit from having expanded access to voice and video chat. If a team of 15 people were to pay monthly ($8) and secure an 85% discount, the cost of an annual Standard Slack account would amount to $216, a cost that would be worthwhile if it meant that all communication could take place in and from a central environment.

Overall, the free version of Slack provides an ideal solution for individuals overseeing projects with multiple components and collaborators. The workspace keeps communications and integrated applications in one place, increasing productivity and improving organization by separating conversation topics, creating a searchable archive of content, and allowing team members to save time by reading only conversations that are relevant to them.
